# Anti-inflammatory effect of hydroxyproline-GQDGLAGPK in desiccation stress-induced experimental dry eye mouse

**DOI:** 10.1038/s41598-017-07965-4

**Published:** 2017-08-07

**Authors:** Hyesook Lee, Chae Eun Kim, Byul-Nim Ahn, Jaewook Yang

**Affiliations:** 1 T2B infrastructure center for ocular diseases, Inje University Busan Paik Hospital, 75 Bokji-ro, Busanjin-gu, Busan 47392 Republic of Korea; 2 Department of Ophthalmology, Inje University College of Medicine, 75 Bokji-ro, Busanjin-gu, Busan 47392 Republic of Korea; 3 Eyebio Korea, F 1010, 197 inje-ro, Gimhae-si, Gyeongsangnam-do 50834 Republic of Korea

## Abstract

The purpose of this study has been the investigation of the effect of novel peptide hydroxyproline-GQDGLAGPK (Hyp-GQDGLAGPK) in desiccation stress-induced dry eye mouse model and compared medicines for dry eye disease including cyclosporine, diquafosol and sodium hyaluronate. Seventy eight NOD.B10.*H2* ^*b*^ mice were injected with scopolamine and exposed to an air draft for 10 days, and then the mice were treated with normal saline (n = 13), 1% Hyp-GQDGLAGPK (n = 13), 0.05% cyclosporine (n = 13), 3% diquafosol (n = 13), and 0.1% hyaluronate (n = 13) for 10 days. Thirteen mice were used for histopathologic analysis at DS 10d. The desiccation stress significantly decreased tear production, but the topical treatment of Hyp-GQDGLAGPK recovered to the baseline levels, which was similar to cyclosporine and diquafosol. In addition, Hyp-GQDGLAGPK improved facilitating epithelium stabilization including the corneal irregularity score, fluorescein score and detachment of the corneal epithelium. These improvements in stabilization of the corneal epithelium was superior to that in the cyclosporine and sodium hyaluronate groups. Furthermore, desiccation stress markedly induced expression of autoimmune inflammation-related factors in the lacrimal glands, but it was significantly suppressed by Hyp-GQDGLAGPK treatment. Overall, we found that novel peptide Hyp-GQDGLAGPK has multi-functional effects such as stabilizing the tear film and inhibiting inflammation.

## Introduction

Dry eye is a multifactorial diseases of the tears film and ocular surface characterized by eye discomfort, visual disturbance, tear film instability and chronic inflammation affects an estimated 20 million patients in the United States alone^1, 2^. Factors that disturb the delicate homeostatic balance of the ocular surface can adversely affect tear film stability and osmolarity, resulting in osmotic, mechanical, and inflammatory damage^2^. Recent studies have shown that dry eye is an inflammatory disease with many features in common with autoimmune disease^2–4^. One of the non-obese diabetic (NOD) mouse strains, NOD.B10.*H2* ^*b*^, was found to induce spontaneous dry eye and Sjogren’s syndrome-like autoimmune inflammation in the ocular surface and lacrimal gland and this process can exacerbated by desiccating stress^5, 6^. Desiccating ocular surface stress has been known to stimulate T cell activated autoimmune event that cause Sjőgren’s syndrome-like inflammation^2, 5^. The inflammatory reactions of the ocular surface result in a vicious cycle of damage to the ocular surface, including gradual dysfunction and destruction of the lacrimal glands and impairment of the conjunctival epithelium^7, 8^. Currently, based on the concept that inflammation is a major factor in the pathogenesis of dry eye, anti-inflammatory agents are the current mainstream choice for treating dry eye among the various treatment options^9^.

Cyclosporine exerts immunosuppressive activity and representative anti-inflammatory therapies for dry eye disease (DED)^10^. Topical administration of cyclosporine has been shown to increase tear fluid secretion, goblet cell density and decreases epithelial cell apoptosis and inflammatory cytokines in the conjunctiva and lacrimal glands, but it is clear that many patients with DED do not show a consistent therapeutic response to topical cyclosporine^11–13^. Diquafosol stimulates section of fluid and mucin from the conjunctiva directly on the ocular surface by interacting with the P2Y2 receptors to increase the tear film stability^14^. Fujihara T *et al*. shown that diquafosol promotes tear fluid and mucin secretion, and it suppresses corneal epithelial damage^15, 16^. However, some studies have reported that diquafosol may not act on the lacrimal glands directly, or not accompanied with a major improvement in symptoms related to DED^17–19^. Sodium hyaluronate (hyaluronic acid, hyaluronan) is a linear polymer composed of long chains of repeating disaccharide units of N-acetylglucosamine and glucuronic acid, and is the most characteristic component of synovial fluid^20, 21^. It has a high capacity to retain water and resists desiccation, which improves the wettability of the ocular surface^20–22^. Although sodium hyaluronate has been reported to protect the corneal epithelium and to retain the tear, it has limited function of lacrimal gland and goblet cells^23^.

Extracellular matrix consists of complex mixture of structural and functional proteins including glycosaminoglycans (GAGs), proteoglycans, glycoproteins, and collagens^24^. These components serves an important role in tissue and organ morphogenesis, and induce the surrounding cells to repair the wounded tissue instead of forming scar tissue^24, 25^. The ECM also prevents the triggering of immune responses that are associated with inflammation^26–28^. Our previous reports have shown that chondrocyte-derived ECM (CDECM) suppressed corneal neovascularization (NV) and opacity by modulating the inflammatory reaction in alkaline burn animal models^28, 29^. Furthermore, we reported that CDECM has anti-angiogenic effects in pterygium mouse and suture-induced corneal NV rabbits^30, 31^. In addition, CDECM has improved symptoms of inflammation in a dry eye mouse model^32^. However, we did not establish which components from CDEMC benefit ocular surface diseases. In 2007, Jin *et al*. reported that the composition of the CDECM scaffold consists of 73% collagens, 16% GAGs, and 11% water^33^. Collagen has previously been introduced as a wound-healing agent for treating burns and skin ulcerations^34, 35^. In addition, Wang *et al*. reported that the ECM fragments, the type II collagen-derived N-terminal propeptide, was also shown to have anti-angiogenic activity *in vitro* ^36^. Furthermore, treatment with collagen promotes corneal epithelial and stromal healing in animal and human subjects^37,38]^. Nevertheless, there has been no study for the efficacy evaluation of collagens in DED with inflammation.

On the basis of the concept that CDECM has anti-inflammatory and anti-angiogenic effects on ocular surface diseases, we hypothesize that collagen from CDECM has a better therapeutic effect against inflammation-associated DES. In the present study, we isolated and synthesized collagen type II α1-based novel peptide hydroxyproline-GQDGLAGPK (Hyp-GQDGLAGPK) from CDECM. We evaluated the efficacy of Hyp-GQDGLAGPK on symptoms of DED including tear quantity, stabilization of ocular surface, and inflammation in an experimental dry eye mouse model. Furthermore, we studied the effect of Hyp-GQDGLAGPK in comparison with topical cyclosporine, sodium hyaluronate, and diquafosol.

## Results

### Effects of Hyp-GQDGLAGPK on tear production

**Figure 1:**
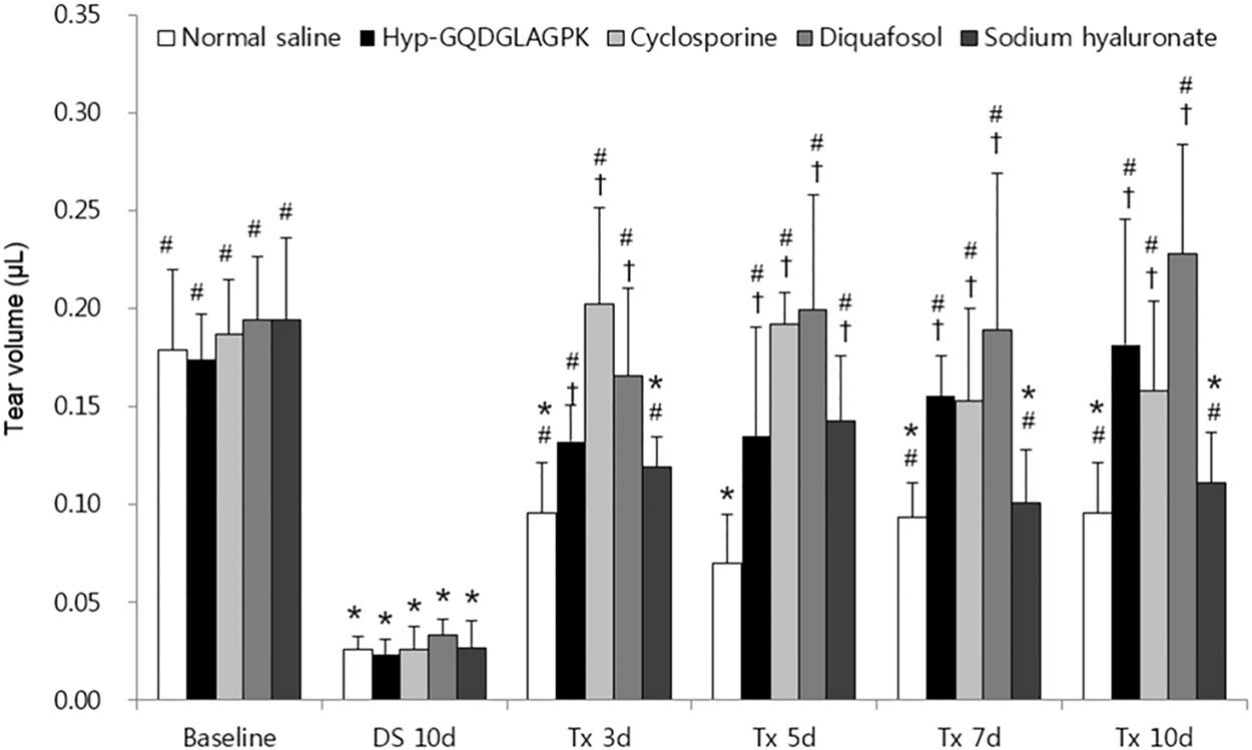
Effect of Hyp-GQDGLAGPK on tear production in the experimental dry eye mouse model. The tear volumes of the Hyp-GQDGLAGPK, cyclosporine, diquafosol, and sodium hyaluronate groups were measured at baseline, after 10 days of desiccation stress (DS 10d), and after treatment for 3, 5, 7, and 10 days. The quantitative data are presented as the means ± SD. The results are statistically significant by analysis of variance with the Tukey test at *p < 0.05 compared with baseline, ^#^ *p* < 0.05 compared with DS 10d and ^†^ *p* < 0.05 vs. corresponding value in the normal saline group. Baseline = before desiccation stress; DS 10d = immediately after desiccation stress for 10 days; Tx 3d, Tx 5d, Tx 7d and Tx 10d = 3, 5, 7 and 10 days after treatment with Hyp-GQDGLAGPK, cyclosporine, diquafosol, and sodium hyaluronate eye drops, respectively.

As shown in Fig. 1, the desiccation stress (0.03 ± 0.01 μL, *p* < 0.0001) significantly decreased tear production compared to baseline (0.18 ± 0.03 μL). The tear volume of the Hyp-GQDGLAGPK group markedly increased to 0.13 ± 0.02 μL (*P* < 0.0001) from 3 days after treatment compared DS 10d. In addition, the topical Hyp-GQDGLAGPK eye drops impaired tear production in a time-dependent manner. After 10 days of treatment, the Hyp-GQDGLAGPK group tear production was 0.18 ± 0.03 μL (*p* = 0.786 vs baseline), which was similar to baseline. At 10 days after treatment, the cyclosporine, diquafosol, and sodium hyaluronate groups also had significantly increased tear volume to 0.16 ± 0.05 μL, 0.23 ± 0.06 μL, and 0.11 ± 0.032 μL compared with DS 10d, respectively (*p* < 0.0001). The normal saline group also significantly increased tear volume (0.10 ± 0.03 μL, *p* < 0.0001) at 10 days after treatment. However, although the tear production of the normal saline group and the sodium hyaluronate group were significantly increased compared with DS 10d, the tear volume did not recover to the baseline levels (*p* = 0.002 vs baseline). Meanwhile, the tear volumes of the Hyp-GQDGLAGPK, cyclosporine and diquafosol groups were statistically different from the normal saline group (*p* = 0.012; *p* = 0.016; *p* < 0.0001, respectively)

### Effects of Hyp-GQDGLAGPK on corneal surface irregularities

**Figure 2:**
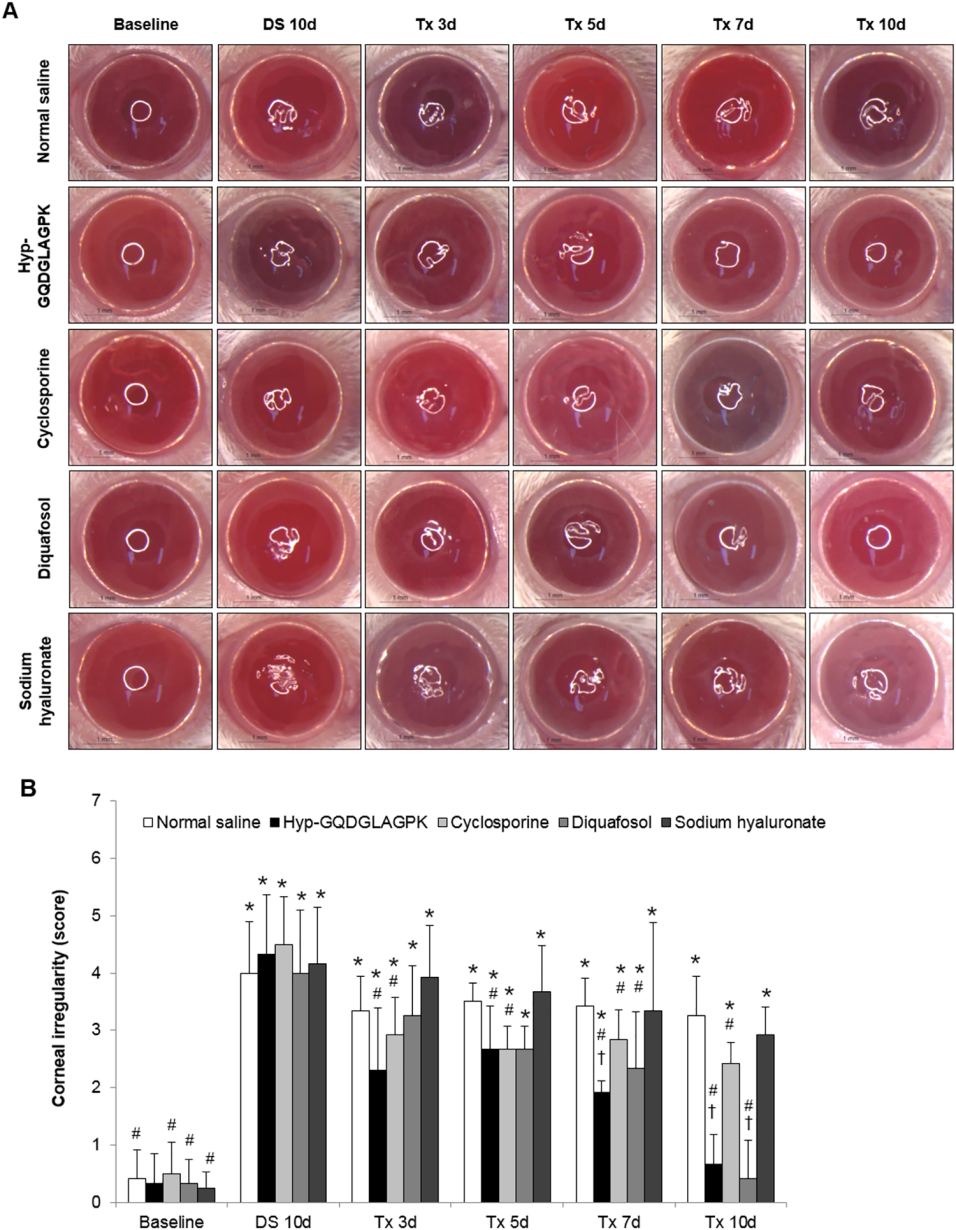
Effects of Hyp-GQDGLAGPK on corneal surface irregularities in the experimental dry eye mouse model. (**A**) Images of the eyes of the Hyp-GQDGLAGPK, cyclosporine, diquafosol, and sodium hyaluronate groups were photographed with a microscope at baseline; after DS 10d; and after treatment for 3, 5, 7, and 10 days. Scale bar = 1 mm. (**B**) The changes in the corneal irregularity scores was measured in the Hyp-GQDGLAGPK, cyclosporine, diquafosol and sodium hyaluronate groups. The quantitative data are presented as means ± SD (n = 13). The results are statistically significant by analysis of variance with the Tukey test at *p < 0.05 compared with baseline, ^#^ *p* < 0.05 compared with DS 10d and ^†^ *p* < 0.05 vs corresponding value in the normal saline group. Baseline = before desiccation stress; DS 10d = immediately after desiccation stress for 10 days; Tx 3d, Tx 5d, Tx 7d, and Tx 10d = 3, 5, 7 and 10 days after treatment with Hyp-GQDGLAGPK, cyclosporine, diquafosol, and sodium hyaluronate eye drops, respectively.

The desiccation stress gradually increased corneal irregularity in all groups (Fig. 2A). However, the Hyp-GQDGLAGPK and cyclosporine groups had a circular white ring from day 3 after treatment, and the diquafosol group had a circular white ring from day 7 after treatment. The distorted white ring of the normal saline and sodium hyaluronate groups did not improve at 10 days of treatment. The quantitative data of corneal irregularity score is shown in Fig. 2B. The irregularity scores following desiccation stress gradually increased to 4.20 ± 0.97 (*p* < 0.0001) compared to baseline (0.37 ± 0.45). In the Hyp-GQDGLAGPK and cyclosporine groups, the scores of corneal irregularity were significantly decreased to 2.31 ± 1.09 and 2.31 ± 1.09 at 3 days after treatment, respectively (*p* = 0.008 vs DS 10d; *p* = 0.005 vs DS 10d). Eye drops of Hyp-GQDGLAGPK markedly decreased the corneal irregularity score to 0.67 ± 0.52 as compared to baseline at 10 days after treatment in an experimental dry eye mouse. The irregularity score of the diquafosol group was gradually decreased to 2.673 ± 0.41 at 5 days after treatments (*p* = 0.019 vs DS 10d), and it was improved to the baseline score at 10 days after treatment (p < 0.0001 vs DS 10d). The corneal irregularity score of the normal saline was statistically different than that at DS 10d (p = 0.134 vs DS 10d).

### The effect of the Hyp-GQDGLAGPK on corneal fluorescein staining

**Figure 3:**
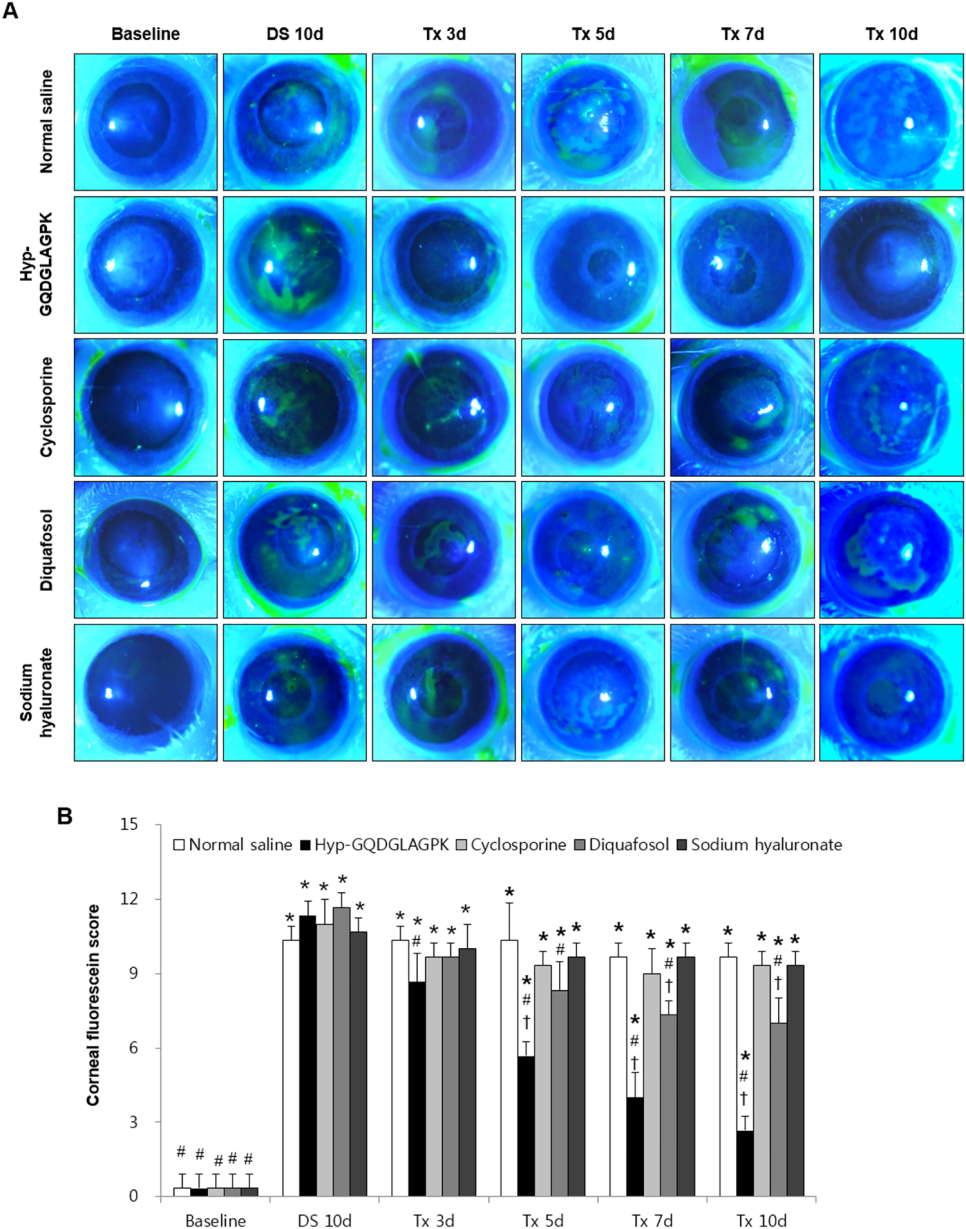
Effect of *Hyp-GQDGLAGPK* on corneal fluorescein staining. (**A**) Fluorescent slit-lamp photographs of the eyes of the Hyp-GQDGLAGPK, cyclosporine, diquafosol, and sodium hyaluronate groups were photographed with a microscope at baseline; after DS 10d; and after treatment for 3, 5, 7, and 10 days. (**B**) Corneal fluorescein grading score at each time point. The quantitative data are presented as means ± SD (n = 13). The results are statistically significant by analysis of variance with the Tukey test at *p < 0.05 compared with baseline, ^#^ *p* < 0.05 compared with DS 10d and ^†^ *p* < 0.05 vs corresponding value in the normal saline group. Baseline = before desiccation stress; DS 10d = immediately after desiccation stress for 10 days; Tx 3d, Tx 5d, Tx 7d, and Tx 10d = 3, 5, 7 and 10 days after treatment with Hyp-GQDGLAGPK, cyclosporine, diquafosol, and sodium hyaluronate eye drops, respectively.

The fluorescein staining of the corneas was significantly increased to 11.00 ± 0.66 by desiccation stress (Fig. 3, *p* < 0.0001 vs baseline). However, eye drops of Hyp-GQDGLAGPK significantly decreased the score of fluorescein staining from 3 days after treatment (8.67 ± 1.16, *p* = 0.023 vs DS 10d), and suppressed to 2.67 ± 0.58 at 10 days after treatment (*p* < 0.0001 vs DS 10d). At 5 days after treatment, the score of fluorescein staining also decreased by diquafosol (8.33 ± 1.15, *p* = 0.011 vs DS 10d), and in time-dependent manner for 10 days. However, although the eye drops of diquafosol improved the score of fluorescein staining as reflection of corneal injury, the score was just 46.15% of efficacy of Hyp-GQDGLAGPK.

Meanwhile, topical eye drops of cyclosporine and sodium hyaluronate did not changed the score of fluorescein staining at each time point.

### Effects of Hyp-GQDGLAGPK on the detachment of corneal epithelial cells

**Figure 4:**
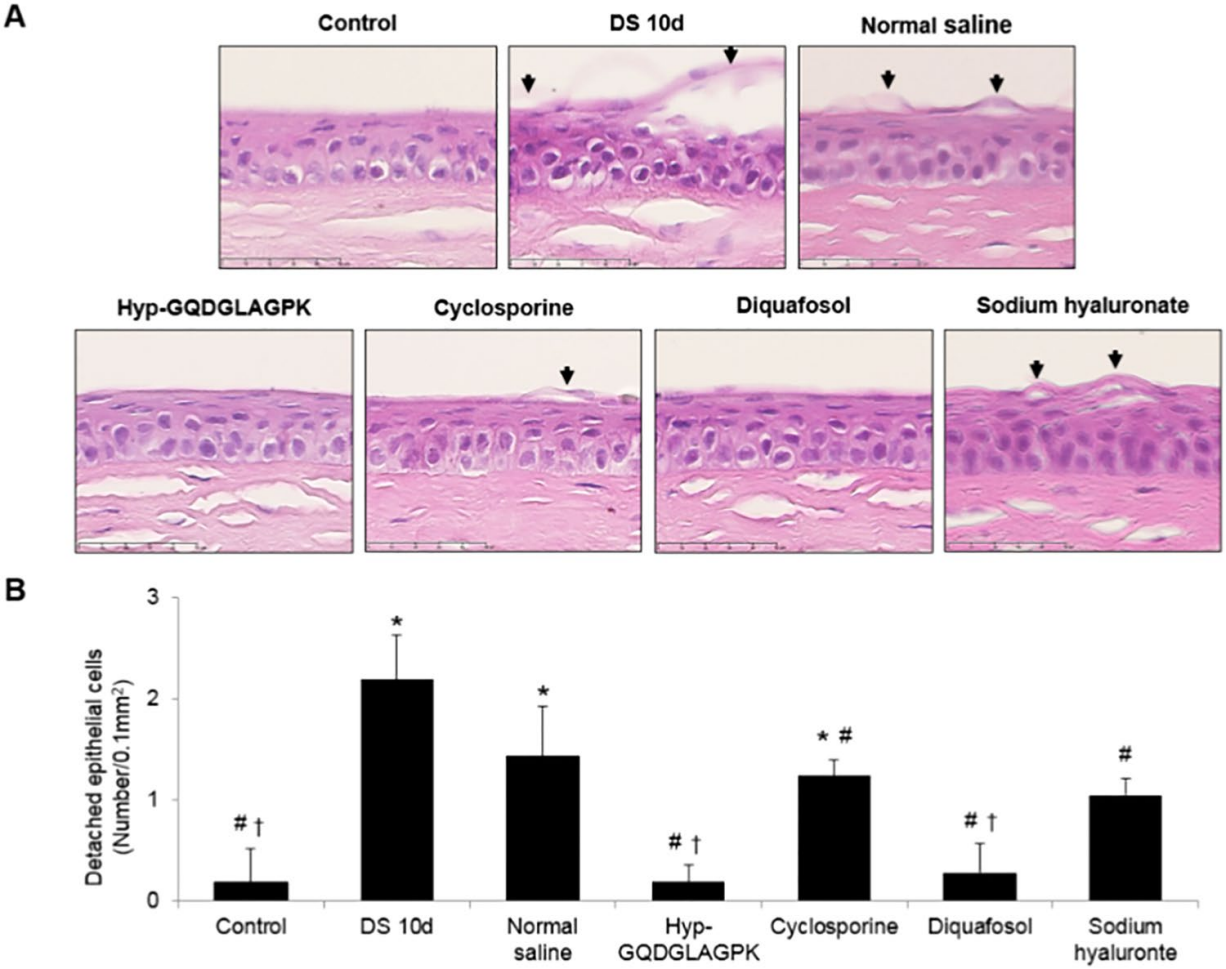
Effect of Hyp-GQDGLAGPK on the detachment of corneal epithelial cells. (**A**) The corneas of the NOD.B10.*H2* ^b^ mice were stained with H&E before desiccation stress, immediately after desiccation stress for 10 days, and 10 days after treatment with Hyp-GQDGLAGPK, cyclosporine, diquafosol, and sodium hyaluronate eye drops. The arrows indicate detached corneal epithelial cells. Scale bar = 50 μm. (**B**) The numbers of detaching corneal epithelial cells are expressed as the means ± the SD (n = 4~5 eyes). The results are statistically significant by analysis of variance with the Tukey test at *p < 0.05 compared with baseline, ^#^ *p* < 0.05 compared with DS 10d and ^†^ *p* < 0.05 vs corresponding value in the normal saline group.

We have performed H&E staining to measure of corneal epithelium detachment. Detached epithelial cells were more frequently observed on DS 10d (Fig. 4A), but the Hyp-GQDGLAGPK, cyclosporine, diquafosol, and sodium hydrate groups showed a reduction in detached corneal epithelium. As shown in Fig. 4B, the quantitative data of detached epithelial cells are indicated as number per 0.1 mm^2^. The desiccation stress gradually increased the detachment of corneal epithelium (*p* = 0.003 vs control) to 2.19 ± 0.44/0.1 mm^2^. This alteration by desiccation stress was not decreased by normal saline (1.43 ± 0.49/0.1 mm^2^, *p* = 0.116 vs DS 10d). However, the detachment of corneal epithelial cells were significantly suppresed by Hyp-GQDGLAGPK to 0.19 ± 0.16/0.1 mm^2^ (*p* = 0.002 vs DS 10d), and this level was similar to that of control. The number of detached epithelial cells in the cyclosporine, diquafosol, and sodium hydrate groups also reduced to 1.24 ± 0.16/0.1 mm^2^, 0.29 ± 0.29/0.1 mm^2^, and 1.05 ± 0.16/0.1 mm^2^, respectively. The detachment of corneal epithelial cells was primarily improved in the Hyp-GQDGLAGPK group, and its efficacy was similar to that of the control and diquafosol groups.

### Effects of Hyp-GQDGLAGPK on conjunctival goblet cells

**Figure 5:**
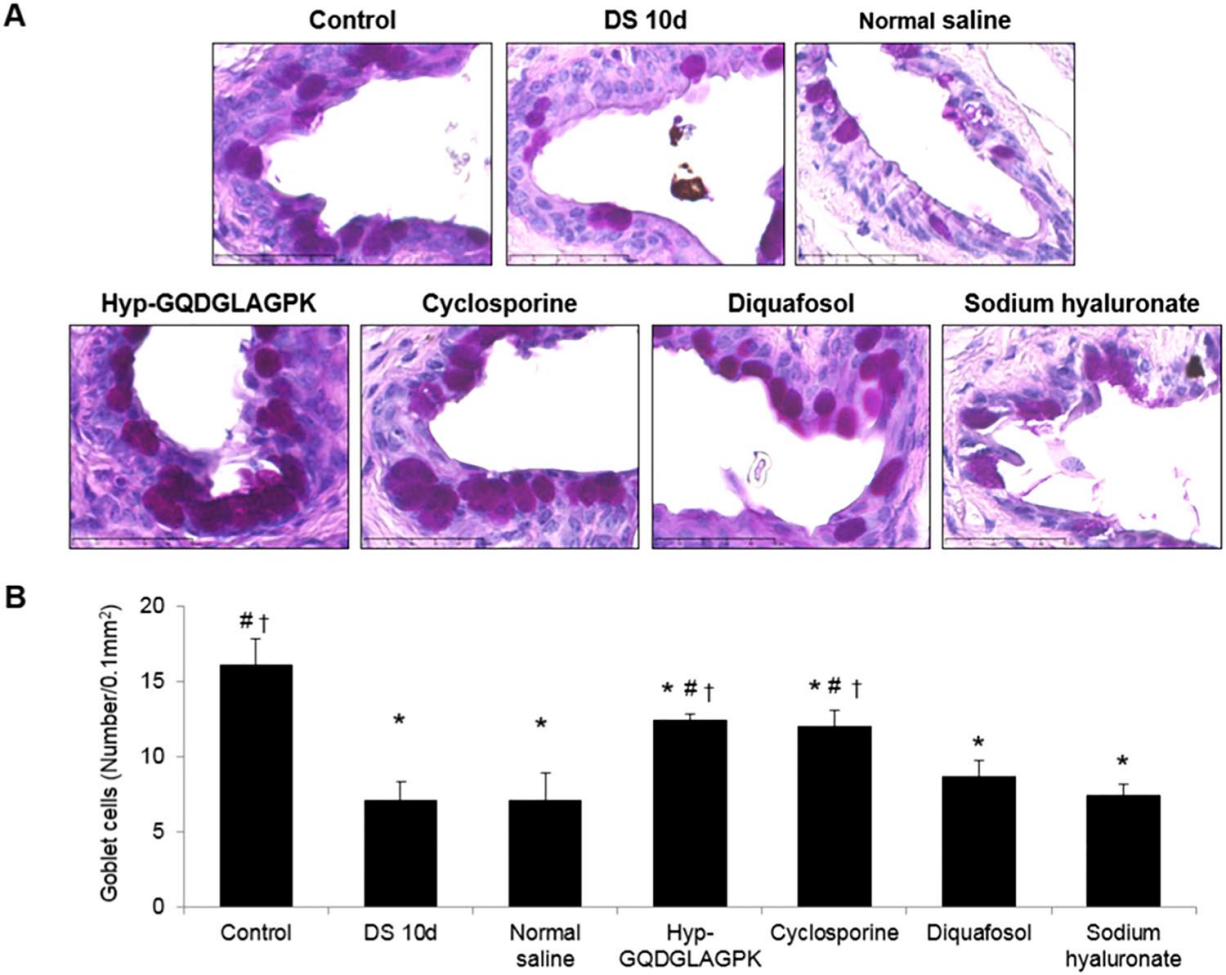
Effect of Hyp-GQDGLAGPK on conjunctival goblet cell densities in the experimental dry eye mouse model. (**A**) The goblet cells of the conjunctiva were stained with PAS before desiccation stress, immediately after desiccation stress for 10 days, and 10 days after treatment with Hyp-GQDGLAGPK, cyclosporine, diquafosol, and sodium hyaluronate eye drops. Scale bar = 50 μm. (**B**) The goblet cell densities of each group are presented as means ± SD (n = 4~5 eyes). The results are statistically significant by analysis of variance with the Tukey test at *p < 0.05 compared with baseline, ^#^ *p* < 0.05 compared with DS 10d and ^†^ *p* < 0.05 vs corresponding value in the normal saline group.

The number of goblet cells was measured in the inferior fornix conjunctiva, as shown in Fig. 5. The number of goblet cells in DS 10d group was significantly decreased to 7.05 ± 1.29/0.1 mm^2^ (*p* = 0.002) compared with control (16.10 ± 1.72/0.1 mm^2^). The eye drops of normal saline, diquafosol and sodium hyaluronate did not increase for 10 days of treatment (*p* = 0.681 vs DS 10d). In contrast, the amount of goblet cells in the Hyp-GQDGLAGPK group and cyclosporine group was significantly improved compared to the number in the DS 10d group (12.38 ± 0.44/0.1 mm^2^, *p* = 0.002 vs DS 10d; 12.00 ± 1.03/0.1 mm^2^, *p* = 0.006 vs DS 10d, respectively).

### Effects of Hyp-GQDGLAGPK on expression of CD4^+^ T-cells

**Figure 6:**
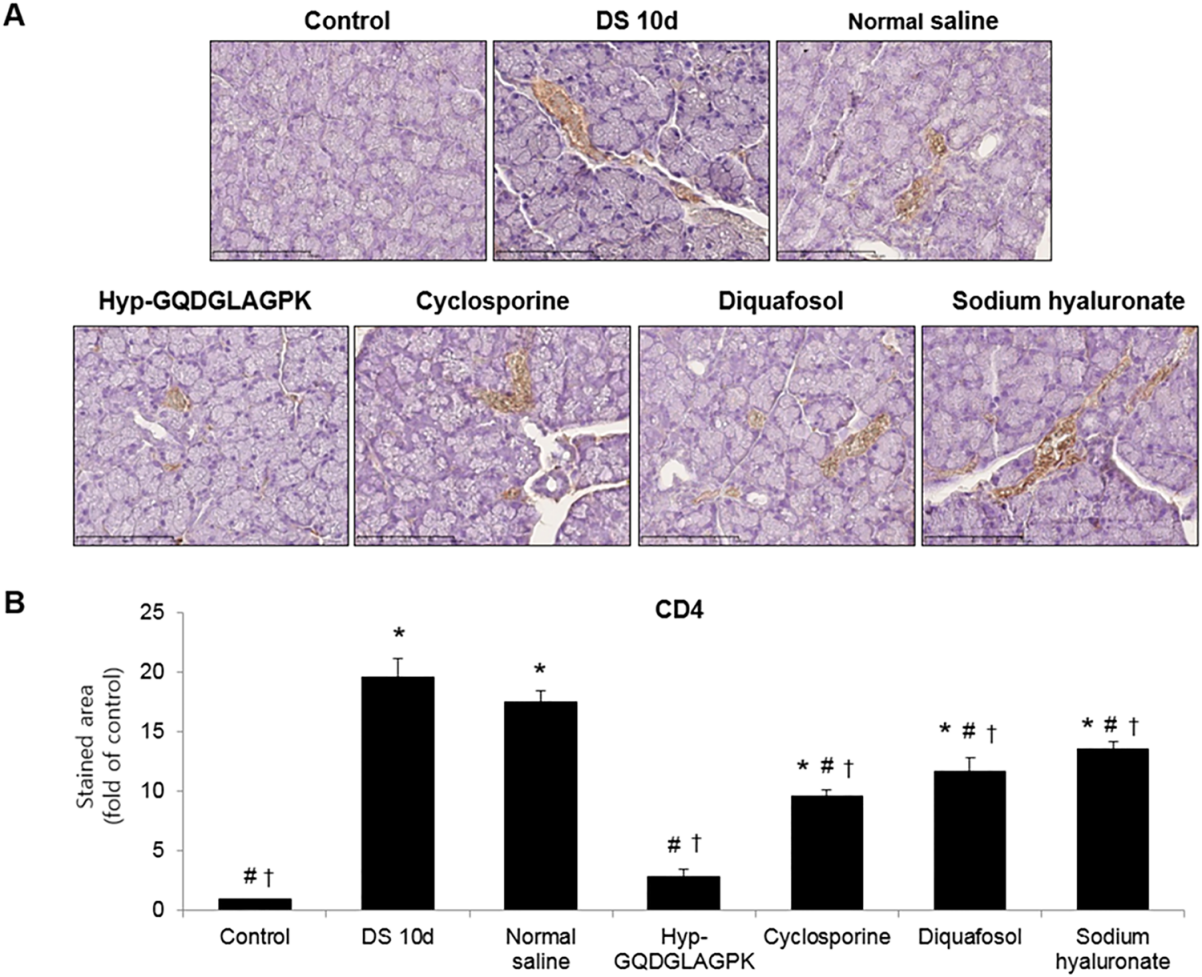
Effect of GQDGLAGPK on expression of CD4^+^ T cells in the lacrimal glands of experimental dry eye mouse model. (**A**) The sections were immunostained with specific antibodies for CD4 before desiccation stress, immediately after desiccation stress for 10 days, and 10 days after treatment with Hyp-GQDGLAGPK, cyclosporine, diquafosol, and sodium hyaluronate eye drops. Scale bar = 100 μm. (**B**) The stained area of the photograph was analyzed using ImageJ® and calculated in terms of the fold of the control. Values are mean ± SD (n = 5~6 eyes). The results are statistically significant by analysis of variance with the Tukey test at *p < 0.05 compared with baseline, ^#^ *p* < 0.05 compared with DS 10d and ^†^ *p* < 0.05 vs corresponding value in the normal saline group. Con = control; DS 10d = immediately after desiccation stress for 10 days; NS = normal saline; 10mer = Hyp-GQDGLAGPK; Cys = cyclosporine; Diq = diquafosol; HA = sodium hyaluronate; CD4 = cluster of differentiation 4.

We assessed the efficacy of Hyp-GQDGLAGPK on the cluster of differentiation 4 (CD4) expression in mice with DED. Figure 6 shown that the desiccation stress markedly upregulated expression of CD4^+^ T-cells (19.53 fold of control, *p* < 0.0001), but it was significantly suppressed by all treatment. The expression of CD4^+^ T-cells was gradually down-regulated by Hyp-GQDGLAGPK (*p* < 0.0001 vs DS 10d), which was similar to control group. The eye drops of cyclosporine markedly decreased expressions of CD4^+^ T-cells, but the efficacy was lower than treatment of Hyp- GQDGLAGPK on expression of CD4^+^ T-cells. Although the expressions of CD4^+^ T-cells were not significantly different between diquafosol and sodium hyaluronate groups, the levels were statistically different compared with DS 10d. The eye drops of normal saline did not decreased expression of CD4^+^ T-cells for 10 days of treatment.

### Effects of Hyp-GQDGLAGPK on the inflammation of lacrimal gland

**Figure 7:**
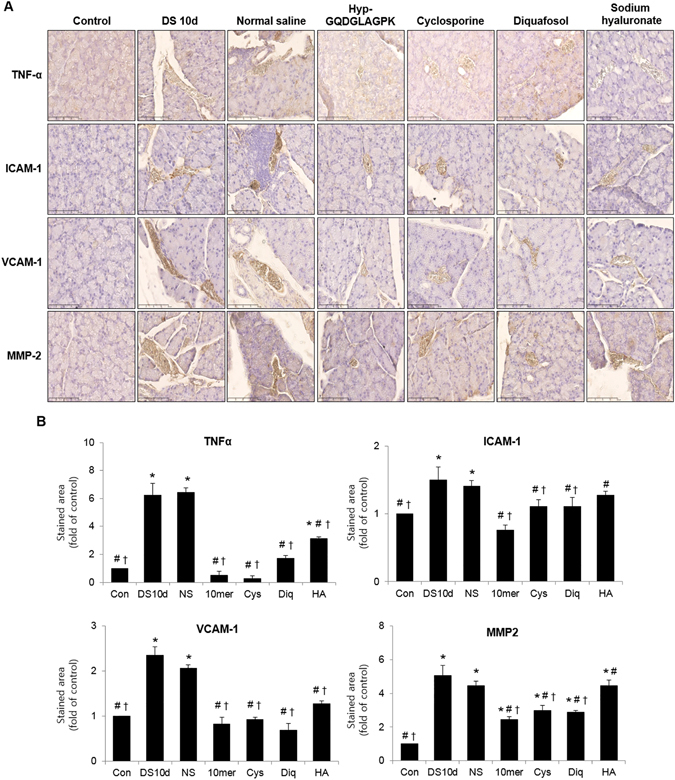
Effect of Hyp-GQDGLAGPK on inflammatory markers in the lacrimal glands of experimental dry eye mouse model. (**A**) The sections were immunostained with specific antibodies for TNF-α, ICAM-1, VCAM-1, and MMP2 before desiccation stress, immediately after desiccation stress for 10 days, and 10 days after treatment with Hyp-GQDGLAGPK, cyclosporine, diquafosol, and sodium hyaluronate eye drops. Scale bar = 100 μm. (**B**) The stained area of the photograph was analyzed using ImageJ® and calculated in terms of the fold of the control. Values are mean ± SD (n = 8~10 eyes). The results are statistically significant by analysis of variance with the Tukey test at *p < 0.05 compared with baseline, ^#^ *p* < 0.05 compared with DS 10d and ^†^ *p* < 0.05 vs corresponding value in the normal saline group. Con = control; DS 10d = immediately after desiccation stress for 10 days; NS = normal saline; 10mer = Hyp-GQDGLAGPK; Cys = cyclosporine; Diq = diquafosol; HA = sodium hyaluronate; TNF-α = tumor necrosis factor alpha; ICAM-1 = intercellular adhesion molecule-1; VCAM-1 = vascular cell adhesion molecule-1; MMP-2 = matrix metalloproteinase 2.

We assessed the effect of Hyp-GQDGLAGPK on the expression of inflammatory factors in mice with DED. The sections of the lacrimal glands of these mice were immunostained with pro-inflammatory specific markers such as tumor necrosis factor alpha (TNFα), intercellular adhesion molecule (ICAM)-I, vascular cell adhesion molecule (VCAM)-1 and matrix metalloproteinase (MMP)-2. As shown in Fig. 7, desiccation stress markedly induced 6.24-fold (*p* < 0.0001 vs control) expression of pro-inflammatory cytokine TNFα in the lacrimal gland compared with that in control, but it was gradually suppressed by all treatments. The Hyp-GQDGLAGPK and cyclosporine groups showed suppression of TNFα expression, and its expression level was similar to that in the control group. The expression of adhesion molecule ICAM-1 also significantly increased by desiccation stress (1.50-fold of control, *p* = 0.009 vs control), but it was markedly decreased by Hyp-GQDGLAGPK eye drops (0.76-fold of control, *p* = 0.0003 vs DS 10d). The expression of another adhesion molecule VCAM-1 in the lacrimal gland was gradually suppressed by all treatments that contained Hyp-GQDGLAGPK eye drops. The expression of MMP2 was significantly increased to 5.07-fold (*p* = 0.001 vs control) of control following desiccation stress, but it was markedly reduced in the Hyp-GQDGLAGPK group (2.44-fold of control, *p* = 0.002 vs DS 10d). Nevertheless, the sodium hyaluronate group did not show suppressed expression of ICAM-1 and MMP2 (respectively, *p* = 0.114 vs DS 10d; *p* = 0.201 vs DS 10d). In addition, the expression levels of all pro-inflammatory markers were not improved in the normal saline group.

## Discussion

In this study, we evaluated the efficacy of collagen type II α1-based novel peptide Hyp-GQDGLAGPK on symptoms of dry eye including tear quantity, stabilization of ocular surface, and inflammation in a DED mouse model. Additionally, we evaluated the effects of Hyp-GQDGLAGPK in comparison with cyclosporine, diquafosol, and sodium hyaluronate. In 2011, Yoon, *et al*. reported that experimental dry eye was developed by subcutaneous injection of scopolamine with exposures to an air draft for 10 day in NOD.B10.*H2* ^*b*^ ^39^. This study suggested that none of the parameters, including tear production, corneal smoothness, conjunctival goblet cells, CD4^+^ T-cell densities and cytokines levels, recovered to baseline levels during a period of 4 weeks after the removal of desiccating stress^39^. Likewise, in our study, the desiccation stress significantly decreased tear production compared to baseline, but the topical Hyp-GQDGLAGPK increased tear production to baseline after 10 days of treatment (Fig. 1). The topical cyclosporine, diquafosol, and sodium hyaluronate groups also showed significantly increased tear production, but the sodium hyaluronate group did not recover to the baseline levels. In addition, the desiccation stress gradually increased corneal irregularity, corneal fluorescein score and detachment of corneal epithelium, whereas it was markedly inhibited by Hyp-GQDGLAGPK as control levels after 10-days treatment (Figs 2, 3 and 4). These improvements for stabilization of corneal epithelium were superior to the cyclosporine and sodium hyaluronate groups. Several studies suggested that the detaching epithelial cells associated with stabilization of ocular surface in dry eye^9, 32, 40^. Pflugfelder *et al*.^40^, Kim *et al*.^32^ and Oh *et al*.^9^ also reported the result of corneal epithelial cell detachment using H&E staining in experimental dry eye mouse model. Therefore, we suggested detached epithelial cells as one of the parameter for ocular surface stabilization using H&E staining.

Mucin is thought to play a very important role in tear film stability^41^. Conjunctival goblet cells secrete the MUC5AC that stabilizes the tear film and decreases its surface tension. Goblet cell loss in dry eyes is often associated with a poorly protected and irregular cornea and may lead to sight-threatening corneal ulceration and perforation^41–43^. Goblet cell densities are thought to be very sensitive indicators of ocular surface disease^44^. In this study, we reported that the number of goblet cells following desiccations stress was significantly decreased, but the numbers of goblet cells in Hyp-GQDGLAGPK, cyclosporine, and diquafosol groups were significantly increased (Fig. 5). However, the eye drops of sodium hyaluronate did not increase for 10 days of treatment.

Inflammation has a prominent role in the development and propagation, and clinical symptoms of dry eye may be dependent on T-cell activation and CD4^+^ T cells are thought to be the primary effector T cells of DED^2, 45^. In the present, we investigated the expression of CD4 + T-cells in lacrimal gland in an experimental dry eye mouse model. The desiccation stress markedly upregulated expression of CD4^+^ T-cells, but it was significantly suppressed by all treatment (Fig. 6). Our result suggested that up-regulated CD4^+^ T cells induced immune/inflammatory response in DED mice. In mouse models, the lacrimal and submandibular glands are the first affected in the disease process, and several inflammatory mediators such as interleukin (IL)-1β, IL-6, IL-17, interferon-γ, TNF-α, chemokine ligand 2, and MMPs have been implicated in DED-associated inflammation^46^. TNF-α, as well known inflammatory cytokine, is involved in immune and inflammatory responses, and recently reported that inhibition of TNF-α can improve DED^47^. We also previously reported that desiccating stress promote the expression of TNF-α in lacrimal gland in NOD.B10.*H2* ^*b*^ ^9, 32^. In addition, cell adhesion molecules including ICAM-1 and VCAM-1 accelerated the infiltration of immune cells into the ocular surface of DED patients. Upregulated cell adhesion molecules have been identified in lacrimal gland of DED patients^48–50^. Matrix metalloproteinases are endopeptidases involved in tissue remodeling, and are produced by hypeosmolar stress in corneal epithelial cells^51^. Furthermore, elevated levels of MMP9 have been identified in mice^9, 32, 52^ and patients with DED^53, 54^. Therefore, we investigated the effects of Hyp-GQDGLAGPK on the expression of inflammatory factors in lacrimal glands from a DED mouse model (Fig. 7). The desiccation stress markedly induced expression of pro-inflammatory-related factors, including TNFα, ICAM-1, VCAM-1, and MMP-2 in the lacrimal glands, but it was significantly suppressed by Hyp-GQDGLAGPK treatment. The benefit of Hyp-GQDGLAGPK on inflammation was more similar and/or superior than cyclosporine.

Overall, our results showed that Hyp-GQDGLAGPK improved all targets on pathology of DED, such as increasing tear production, facilitating epithelium stabilization, and increasing goblet cells, as well as decreasing inflammatory markers in the lacrimal gland. On the other hand, cyclosporine, an anti-inflammatory reagent, induced improvement of tear production, increased goblet cells and decreased inflammatory markers, but had inadequate capacity to stabilize the epithelium. In the topical diquafosol group, the benefits of diquafosol, including tear production, epithelium stabilization, and goblet cells, were similar to Hyp-GQDGLAGPK, but suppression of inflammatory markers in the lacrimal gland was lower than that observed for Hyp-GQDGLAGPK. The effect of sodium hyaluronate was the lowest for preventing DED (Table 1). Consequently, we suggested that Hyp-GQDGLAGPK has multi-functional effects that complemented the disadvantage of commercial medicines. Therefore, Hyp-GQDGLAGPK eye drops can be used to treat DED by stabilizing the tear film and inhibiting inflammation.

**Table 1:** Comparing the efficacy of Hyp-GQDGLAGPK and commercial medicines in an experimental dry eye mouse model.

	Hyp-GQDGLAGPK	Cyclosporine	Diquafosol	Sodium hyaluronate
Tear production	+++	+++	+++	+
Epithelium stabilization	+++	+	+++	+
Goblet cells	++	++	−	−
Anti-inflammation	+++	++	+	−

The effects of Hyp-GQDGLAGPK and commercial medicines on tear production, epithelium stabilization, goblet cell density, and expression of inflammatory markers associated with dry eye disease are indicated as −, +, ++ and +++. No effect expressed as −; + indicated significantly different at DS 10d, but no difference with normal saline; ++ indicated significantly different with DS 10d and normal saline group; +++ indicated similar to baseline.

## Materials and Methods

### Preparation of peptide and eye drops

Collagen type II α1-based peptide sequence hydroxyproline-GQDGLAGPK (Hyp- GQDGLAGPK) was synthesized from BioCeltran (Chuncheon, Korea). The 1% Hyp- GQDGLAGPK eye drops were prepared by dissolving in normal saline (JW Pharmaceutical, Seoul, Korea). The topical cyclosporine (Cyporin® N 0.05%), sodium hyaluronate (Hyaluni® 0.1%) obtained from Taejoon Pharm Co., Ltd. (Seoul, Korea) and diquafosol obtained from Santen Pharmaceutical Co., Ltd. (Diquas® ophthalmic solution 3%, Osaka, Japan).

### Animals and experimental procedures

**Figure 8:**
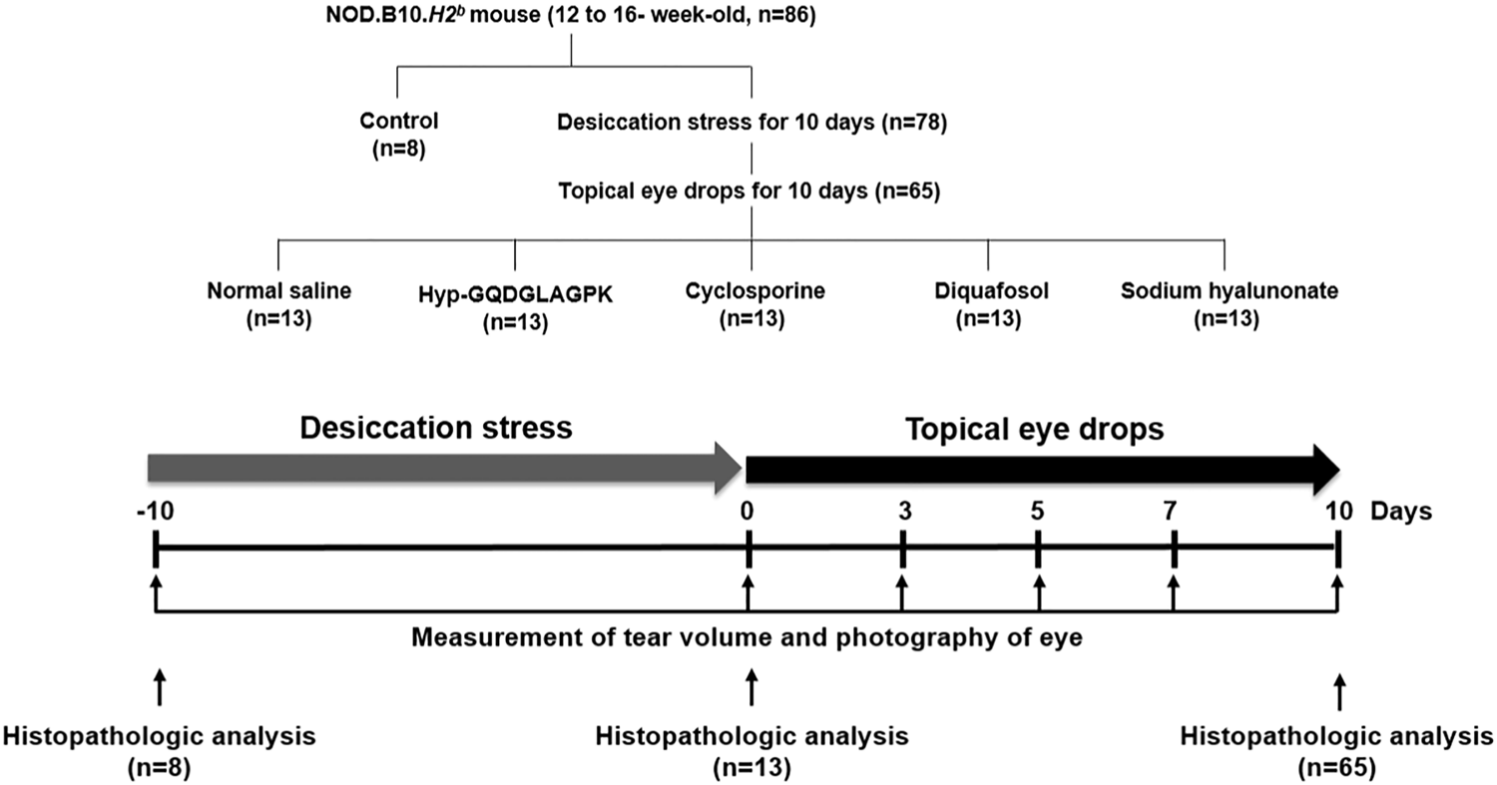
Experimental design of *in vivo* study. Dry eye was experimentally induced in sixty 12- to 16-week-old NOD.B10.H2^b^ mice by subcutaneous injections of scopolamine and exposure to an air draft for 10 days. Ten days later, the mice were randomly divided into five groups: the normal saline group (n = 13, 5 μL of normal saline), the Hyp-GQDGLAGPK group (n = 13, 5 μL of 1% Hyp-GQDGLAGPK), the cyclosporine group (n = 13, 5 μL of Cyporin® N 0.05%), the diquafosol group (n = 13, 5 μL of Diquas® ophthalmic solution 3%), and the sodium hyaluronate group (n = 13, 5 μL of Hyaluni® 0.1%). All treatments were administered five times per day. Tear volume and corneal smoothness were measured at baseline; after 10 days of desiccation stress; and after treatment for 3, 5, 7, and 10 days. Histopathologic analysis was performed before desiccation stress (n = 8, 16 eyes), immediately after desiccation stress for 10 days (n = 13, 26 eyes), and 10 days after treatment with Hyp-GQDGLAGPK, cyclosporine, diquafosol, and sodium hyaluronate eye drops, respectively (n = 13, 26 eyes).

his study was conducted in accordance with a Guideline for Animal Experimentation of Inje University Busan Paik Hospital with approval of the Institutional Animal Care and Use Committee (No.; IJUBPH_2016-005-02) for the use of animals in ophthalmic and vision research. We purchased 86 NOD.B10.*H2* ^*b*^ mice (6 weeks old) from Jackson Laboratory (Bar Harbor, ME, USA). The mice were acclimatized for 6 to 10 weeks. Twelve-week-old or more mice were used for all experiments. Eight mice were used for histopathologic analysis at baseline. Seventy eight mice were subjected to desiccating stress for 10 days that exposed to an air draft with fan as previously reported^9, 32^. At the same time, 0.5 mg/200 μL of the muscarinic receptor blocker, scopolamine hydrobromide, injected to subcutaneous four times a day. Scopolamine hydrobromide obtained from Sigma-Aldrich (St. Louis, MO, USA). On 10 days after desiccating stress (DS 10d), we measured tear amount and corneal irregularity score; tear volume was less than two-thirds of the mean tear volume at baseline and corneal irregularity score ≥ a grade of 2. The dry eye model was successfully established in all mice. Thirteen mice were used for histopathologic analysis at DS 10d. We randomly divided 65 mice into five groups: the normal saline group (n = 13, 5 μL of normal saline), Hyp-GQDGLAGPK group (n = 13, 5 μL of 1% Hyp-GQDGLAGPK), cyclosporine group (n = 13, 5 μL of Cyporin® N 0.05%), diquafosol group (n = 13, 5 μL of Diquas® ophthalmic solution 3%), and the sodium hyaluronate group (n = 13, 5 μL of Hyaluni® 0.1%). Normal saline, Hyp-GQDGLAGPK and sodium hyaluronate administered five times per day for 10 days. Cyclosporine and diquafosol administered 2 times and 6 times per day for 10 day, respectively. Tear amount and corneal irregularity score were measured at baseline, at DS 10d, and after treatment for 3, 5, 7, and 10 days. After treatment for 10, mice were euthanized (Fig. 8).

### Measurement of tear volume

Tear volume was evaluated as previously described^9, 55^. Concretely, the tear amount was evaluated with phenol red–impregnated cotton threads (Zone-Quick; Oasis, Glendora, CA, USA) for 20 seconds. Tear volume was measured at 2 hours after the last scopolamine injection and at 1 hour after the last treatment in both eyes and calculated with a standard curve of response to a stock basic solution (1,500 mL of 0.9% saline and 5 mL of 5 N NaOH).

### Evaluation of corneal irregularity

Corneal irregularity was evaluated as the corneal irregularity score according to the extent of the distortion of the white ring in digital images^56^. The white ring images of the corneal surface were reflected from a fiberoptic circle illuminator with a microscope and were acquired immediately after the mice were euthanized. The corneal irregularity was measured at the same point to tear volume.

### Corneal fluorescein staining

Corneal fluorescein staining was performed according to National Eye Institute (NEI) grading system^57^. Concretely, corneal fluorescein staining was performed according to National Eye Institute (NEI) grading. Ten microliter of 1% fluorescein was applied to the lateral conjunctival sac of the mice, and the eyes were washed by 500 μL of normal saline. Then the eyes were examined for fluorescein staining with a slit lamp biomicroscope (SL-D7; Topcon Medical Systems, Inc., Oakland, NJ) under a cobalt blue light. Punctate staining was recorded in a masked fashion using the standard NEI grading system, giving a score from 0 to 3 (0 = normal and 3 = severe) for each of the five areas (superior, nasal, central, inferior, temporal) of the cornea. Grade 0 is specified when no staining is present, and the maximum score is 15.

### Histology

The orbit of mice was surgically extracted and fixed in 10% formalin. The tissues embedded in paraffin, and were cut to 5 μm with microtome (RM2245, Leica Biosystems, Nussloch, Germany). For the evaluation of the detaching epithelial cells associated with stabilization of ocular surface in DED, the sections were stained with hematoxylin and eosin (H&E)^9, 32^. For the evaluating of conjunctival goblet cells density, the conjunctival sections were stained with periodic acid Schiff (PAS), and performed using a commercial kit (Merck, Darmstadt, Germany) according to the manufacturer’s instructions. The sections were photographed with a virtual microscope (NanoZoomer 2.0 RS, Hamamatsu Photonics, Shizuoka Prefecture, Japan). Goblet cell density in the superior and inferior conjunctiva was measured in three sections of each eye using “threshold tool” of ImageJ®^58^ and was indicated as the number of goblet cells per 100 μm^40^.

### Immunohistochemistry

The lacrimal gland of mice was surgically extracted, fixed in 10% formalin, and embedded in paraffin. Five-micrometer sections were cut with microtome (RM2245). Immunohistochemical analysis of lacrimal gland performed by the method according to Kim *et al*.^32^. The primary antibodies for TNFα and MMP-2 were obtained from Abcam, Inc. (Cambridge, MA, USA). The ICAM-1 antibody and VCAM-1 antibody were purchased from Bioss, Inc. (Woburn, MA, USA). The CD4 antibody was obtained from Novus (Novus Biologicals, LLC., Littleton, CO. USA). Images of the sections were photographed with a virtual microscope (NanoZoomer 2.0 RS). The quantitative analysis of histological staining for CD4, TNFα, ICMA-1, VCAM-1 and MMP-2 performed using “threshold tool” of ImageJ®^58^.

### Statistical analyses

The data were analyzed with SPSS version 22.0 (SPSS, Chicago, IL, USA) and were indicated as means ± standard deviations (SDs). The differences between the groups were analyzed using 1-way ANOVA and statistical significance was defined at *p* < *0.05* by Tukey’s test.
